# Breakthroughs in Cancer Immunotherapy: An Overview of T Cell, NK Cell, Mφ, and DC-Based Treatments

**DOI:** 10.3390/ijms242417634

**Published:** 2023-12-18

**Authors:** Sunyoung Lee, Tae-Don Kim

**Affiliations:** 1 Immunotherapy Research Center, Korea Research Institute of Bioscience and Biotechnology (KRIBB), Daejeon 34141, Republic of Korea; christy0925@kribb.re.kr; 2 Division of Life Sciences, Korea University, Seoul 02841, Republic of Korea; 3 KRIBB School of Bioscience, Korea University of Science and Technology (UST), Daejeon 34113, Republic of Korea

**Keywords:** immunotherapy, cell-based immunotherapy, immune cell, cancer treatment, chimeric antigen receptor, cancer therapy

## Abstract

Efforts to treat cancer using chimeric antigen receptor (CAR)-T therapy have made astonishing progress and clinical trials against hematopoietic malignancies have demonstrated their use. However, there are still disadvantages which need to be addressed: high costs, and side effects such as Graft-versus-Host Disease (GvHD) and Cytokine Release Syndrome (CRS). Therefore, recent efforts have been made to harness the properties of certain immune cells to treat cancer—not just T cells, but also natural killer (NK) cells, macrophages (Mφ), dendritic cells (DC), etc. In this paper, we will introduce immune cell-based cellular therapies that use various immune cells and describe their characteristics and their clinical situation. The development of immune cell-based cancer therapy fully utilizing the unique advantages of each and every immune cell is expected to enhance the survival of tumor patients owing to their high efficiency and fewer side effects.

## 1. Introduction

Despite all the research to overcome cancer, it still shows a high mortality rate and is one of the major challenges for improving overall human survival. For decades, numerous efforts to treat cancer were made with therapies including surgery, radiation, chemotherapy, hormonal therapies, targeted therapies, stem cell therapies, and immune therapies [1]. Among them, immunotherapy significantly improved the survival and the quality of life of cancer patients compared to the previous standard of care [2]. Cancer is characterized by mutations in tumor antigens due to genomic instability. Immunotherapy targets these mutated tumor antigens and has shown high therapeutic efficacy [1]. Indeed, some patients with metastatic cancer, once thought to be incurable, were able to reach long-term remission and be fully cured. For this reason, immunotherapy could be firmly established as the fourth cornerstone of cancer therapy following surgery, radiation, and chemotherapy [1].

There are various types within the category of immunotherapy, including checkpoint inhibitors (ICIs), cancer vaccines, monoclonal antibodies (Abs), immune system modulators, and immune cell-based therapies. In particular, we will focus on the definition and characteristics of immune cell-based immunotherapy (each subtype divided by cell sources), and future research directions.

## 2. T Cell-Based Immunotherapy

### 2.1. Characteristics of T Cells

T cells are one of the predominant adaptive immune cells that recognize specific antigens displayed by major histocompatibility complex (MHC) molecules on tumors or antigen presenting cells (APC), resulting in the death of their targets [3]. Discovering and focusing tumor-specific or over-expressed self-antigens is crucial in order to induce tumor destruction [3]. Due to this ability to recognize and eliminate cancer cells just like cancer-killing soldiers, T cells are by far the most studied among the immune cells. Six CAR-T therapies for B cell malignancies have Food and Drug Administration (FDA) approval [4,5,6], and there are more than 1000 CAR-T clinical trials listed on clinicaltrials.gov.

### 2.2. Tumor-Infiltrating Lymphocyte (TIL)

Tumor-infiltrating lymphocyte (TIL) therapy involves isolating TILs, expanding them, and injecting them back into the patient [3]. The infiltrating autologous T cells isolated from the tumor are co-cultured with antigen-presenting cells (APCs) loaded with antigens. The APCs used for co-culture with T cells are generated from monocytes derived from the patient’s peripheral blood mononuclear cells (PBMCs) and are loaded with specific antigens derived from mutational and immunopeptidome analysis of the patient’s tumor [3].

**Figure 1:**
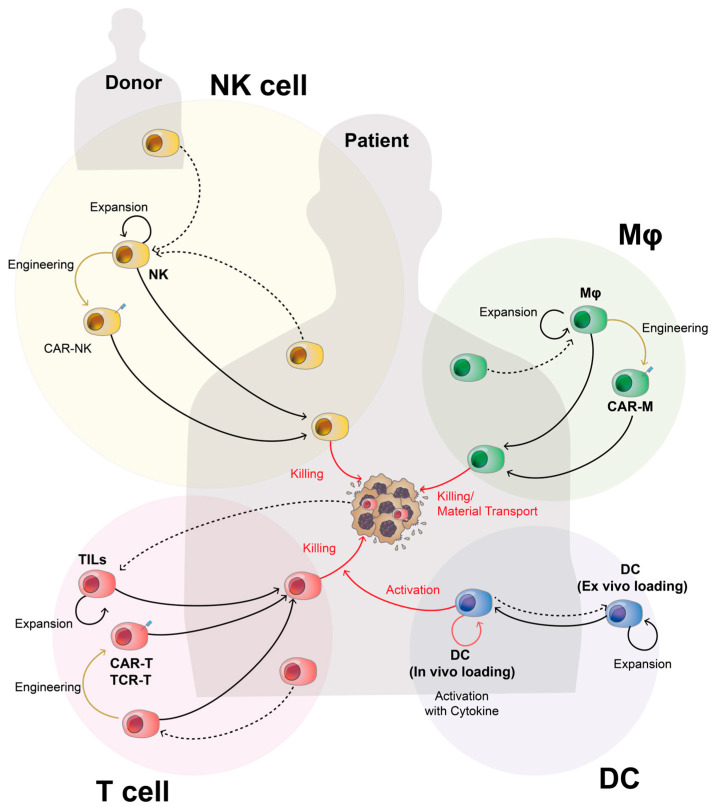
Schematic illustration about the overall flow of cell-based immunotherapy including types of immune cells and their production. In cell-based immunotherapy, the immune cells that will serve as the basis for the live drugs, T cells, natural killer (NK) cells, macrophages (Mφ), and dendritic cells (DCs) are obtained from the patient or the donor, expanded, and activated ex vivo to increase the number of activated cells (dashed arrow). They can also be engineered to express specific antigen recognition receptors, such as CARs or TCRs. The expanded/engineered immune cells are then injected back into the patient to induce tumor killing (solid arrow). Notably, patients could also be injected with cytokines for the activation of DCs without extracting them ex vivo.

TIL therapy entails harvesting T lymphocytes infiltrated by a patient’s tumor, expanding them ex vivo, and using them as a therapeutic agent, as described in Figure 1. Because TILs already have the capacity to recognize the tumors in patients, these lymphocytes, after being expanded ex vivo, could be reinjected into the patient without any modification. There are many different types of lymphocytes from a patient’s tumor, and after obtaining the TILs, the lymphocytes with the most ability for recognizing tumor cells are identified and selected.

Lymphocytes best responding to the tumor are then expanded with cytokines and other substances to be amplified before administration. Unlike CAR-Ts, the target antigen does not need to be identified when using TILs. Despite being successful for specific tumor types like melanoma, this strategy was only possible in surgically resectable tumors that allow sufficient T cells to be harvested and expanded [7].

### 2.3. CAR-T

CAR-Ts are T cells engineered to express Chimeric Antigen Receptors (CARs), which are artificial receptors that recognize antigens of which they already have information. CAR-T therapy is similar to TIL therapy, but unlike TILs, CAR-Ts are engineered to make and express a protein called CAR that is designed to attach to specific proteins on tumor surfaces—to enhance their ability to attack cancer cells—then to be expanded and delivered back to the patient. This process enables CAR-Ts to target and efficiently kill cells expressing the specific antigen. CARs are typically composed of four domains: an antigen-binding domain, a spacer or hinge region, a transmembrane region, and a signaling domain [3]. To improve CAR-T, the CAR domain has evolved. The first generation of CARs contained only ScFv and CD3Z to recognize antigens [8]. After construction of the first-generation CAR, based on the biological understanding that the TCR needs other costimulatory molecules to promote strong signaling, second- and third-generation CARs were constructed with one or two costimulatory domains each. Those second- and third-generation CARs with costimulatory domains have shown stronger antitumor cytotoxicity, increased cytokine production, and improved proliferation and persistence than first-generation CARs, resulting in increased efficacy. Since then, research has continued to design optimized domains to increase efficacy and antigen specificity [8], and strategies to improve the safety of CARs by not only increasing efficacy but also reducing side effects are being explored, including suicide genes, combinatorial target-antigen recognition, synthetic Notch receptors, on-switch CARs, and inhibitory CARs [9].

### 2.4. TCR-T

TCR-Ts are T cells designed to present T cell receptors (TCRs) which target an antigen they already have information about. Because TCR-Ts take advantage of the TCR’s ability to recognize all epitopes presented by the major histocompatibility complex (MHC), the repertoire of antigens they can recognize is more diverse than that of CAR-Ts. In addition, the epitope densities needed to trigger activation are smaller for TCR-T cells than conventional CAR-T cells, so they possess higher antigenic sensitivity. This results in enhanced efficiency in detecting and killing tumor cells [10,11]. Finally, the more aggressive the TCR-T cell, the better the efficacy, and the lower the affinity of TCRs for the target compared to CARs, the more the TCR-T cell can “scan” and remove tumor cells with multiple antigens.

Therefore, TCR-T has gained prominence as an alternative to CAR-T therapy. However, antigen recognition by TCR-T is limited to epitope-presenting Human Leukocyte Antigen (HLA) alleles, the possibilities being not open to all patients.

### 2.5. Clinical Trial

**Figure 2:**
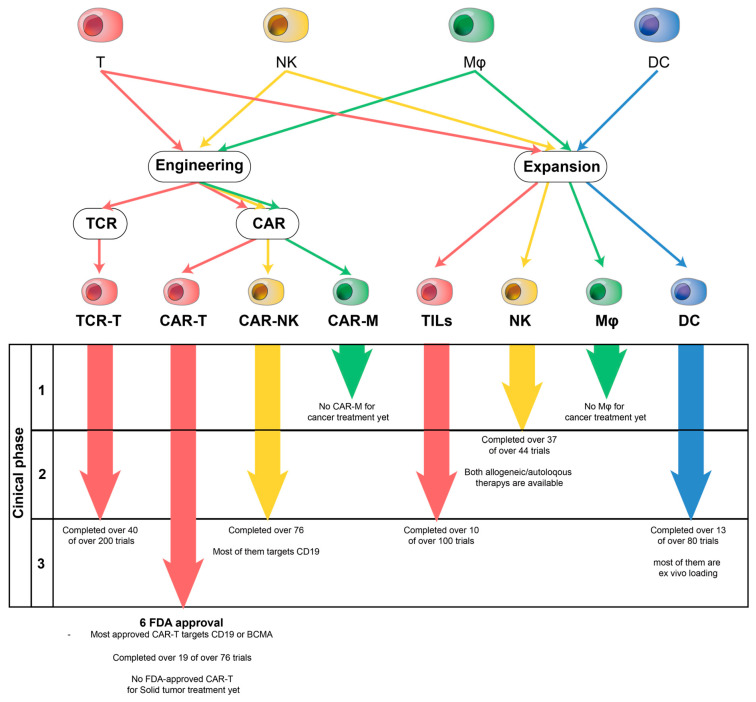
Illustration depicting the progress of clinical trials and representative features of each immune cell therapy. T-cell-based immune cell therapies (TCR-T, TILs, CAR-T) have undergone the most clinical trials, with six CAR-Ts receiving FDA approval. Among autologous, allogeneic NK cell transfer, and CAR-NK, only CAR-NK has reached phase II clinical trials. Both CAR-T and CAR-NK primarily target CD19 and BCMA. There are more than 80 ongoing trials on DCs, mostly in combination with other drugs. Mφ targets a wide range of diseases, but neither Mφ nor CAR-M has been tested for cancer-specific therapies.

For each cell-based therapy, we searched https://clinicaltrials.gov/ (7 November 2023) for completed clinical trials to date. To date, over 1000 CAR-T clinical trials have been enrolled. Among them, a total of 76 CAR-T therapies have been completed, 19 of which have completed clinical phase 2, as described in Figure 2. Most of the clinical trials targeted B cell-based hematopoietic malignancies, with 12 studies targeting CD19 and 5 studies targeting B-cell Maturation Antigen (BCMA). Six of these have been approved by the FDA. In addition, a phase 1 study to increase recognition efficiency with CD19/CD20 dual-CAR-T (NCT04260945), which recognizes both CD19 and CD20, has been completed. Also, a study targeting CD7, an antigen on T cells, to treat T-ALL (NCT04572308) has been completed through phase 1.

Several clinical trials targeting solid tumors (STs) as well as hematopoietic malignancies have been enrolled and completed. Two among the studies using CAR-Ts for treatment of STs have completed clinical phase 2. One of them targeted breast tumors using PD-1 Knockout Anti-MUC1 CAR-T Cells (NCT05812326), and the other treated glioblastoma by CAR-T targeting EGFR (NCT01454596). In addition, there are other studies on hepatocellular carcinoma (NCT03884751, NCT01837602, NCT02416466, NCT03980288, NCT02850536, NCT02395250, NCT02395250, NCT03146234, and NCT02905188), neuroblastoma and glioblastoma (NCT02761915 and NCT01109095), and pancreatic ductal adenocarcinoma (PDA) (NCT01897415), which have also completed phase I studies in STs. 

More than 200 clinical trials using TCR-Ts have been enrolled, and more than 40 have completed Phase II [12,13,14]. These include studies on a variety of cancers, including Acute Myeloid Leukemia (AML) (NCT02550535), myeloma (NCT01352286), Acute Lymphoblastic Leukemia (ALL) (NCT01810120), ovarian cancer (NCT01567891), and breast cancer (NCT01967823 and NCT02111850).

More than 100 clinical trials utilizing TILs have been enrolled and more than 10 have completed Phase II. Ongoing studies with TILs focus on combination therapy with a variety of drugs, including PEG-interferon, vemurafenib, and checkpoint inhibitors such as pembrolizumab (NCT02379195, NCT02354690, NCT03287674, and NCT02500576).

### 2.6. Limitations

Despite that CAR-T therapies showed significant treatment efficacy for hematologic malignancies, the results of clinical experience for almost five years since the first CAR-T cell products were market-approved have not been so positive. The current generation of CAR-T did not achieve long-lasting response in most patients, with a large portion of treated patients ultimately experiencing disease relapse and death [15]. In addition, because T cell therapy utilizes autologous cells, it is complex and currently expensive to manufacture. It can also cause side effects such as Cytokine Release Syndrome (CRS) and Graft-versus-Host Disease (GvHD), which is life-threatening. The success of T cell therapy could also depend on numerous other factors. For instance, the tumor microenvironment can affect T cell function and promote immune evasion [3,15]. Because of this, it is more difficult to establish CAR-T cell therapies targeting STs than hematopoietic malignancies. To date, no CAR therapies have yet been approved for STs [4]. To address this, better understanding of the organic relationship between T cells and the immune system is crucial.

Also, there is a need for combination therapies that target both cancer cells and the immune system [3]. Finally, addressing remaining challenges, such as limited specificity, persistence, and toxicity is also essential for the development of more efficient and safer CAR-T therapy.

## 3. NK Cell-Based Immunotherapy

### 3.1. Characteristics of NK Cells

Natural Killer (NK) cells are principal innate immune cells that protect organisms by destroying transformed targets. They detect various kinds of activating/inhibitory ligands expressed on the target cell, take up signals into the cell, integrate them, and decide whether to kill the target cell. When it decides to kill, the NK cell secretes perforin and granzyme across the immune synapse between the target and itself to induce killing. While both NK cells and T cells have cytotoxic properties, NK cells appear to be more important in early tumor elimination than T cells.

NK cells as live drugs have several powerful advantages. First, NK cells are safe, as they do not have adverse effects such as CRS and GvHD, and they have great off-the-shelf utility. In addition, allogeneic cell therapy can be performed with donors other than the recipients themselves, which can lower the cost. These properties of NK cells could compensate for the drawbacks of T cell-based therapies, making them a universal, safe, and the potent candidate to replace T cells as live drugs. NK cell therapies include stimulating NK cells obtained from patients or donors, delivering them to patients, and injecting CAR-NKs engineered to express CARs [16].

### 3.2. Autologous and Allogenic NK Cell Transfer

NK cells isolated from patients are activated and expanded with various types of cytokines (IL-2, IL-12, IL-15, IL-18, IL-21) in both CAR-dependent and CAR-autologous NK cell transfer [16,17]. After expansion and activation, the NK cells are infused back into the patient, who typically is treated with a cytokine (most often IL-2) to maintain the number and functionality of the injected NK cells. Despite that autologous NK cells could recognize activating signals on tumors, their antitumor activity is limited by inhibitory signals, conveyed from autologous HLA molecules. 

Allogenic NK cell transfer is a method in which NK cells are obtained from the donor, expanded, and activated ex vivo in the same way as an autologous NK cell transfer, and then infused into the patient. When using NK cells expressing Killer cell Immunoglobulin-like Receptor (KIR) molecules which do not recognize the specific HLA molecules of patients due to a KIR-ligand mismatch, the best response is achieved because they do not receive a negative signal.

### 3.3. CAR-NKs

CAR-T therapy is one of the most advanced cellular therapies and has been shown highly effective against blood cancers. However, there still are some challenges. Since CAR-T therapy requires the use of patients’ autologous cells, it is time-consuming to engineer and requires high costs. In addition, it is not easy to obtain autologous T cells from patients to generate CAR-T cells [17,18]. Also, the fundamental side effect of using autologous cells, GvHD, is a critical obstacle. To compensate for these shortcomings of CAR-T cell therapy, CAR-NKs have emerged as an attractive alternative. Unlike CAR-T cells, CAR-NK cells require less time to produce, are less expensive, and are easier to supply because NK cells can be obtained from a variety of sources including peripheral blood (PB), umbilical cord blood (CB), induced pluripotent stem cells (iPSCs), and NK cell lines [19,20]. Another advantage is that CAR-NK cells can use both CAR-dependent and CAR-independent NK-mediated cell killing, providing a stronger safety profile compared to T cell competitors [4,15].

To make CAR-NK cells, NK cells are engineered to display CARs that sense tumor-specific antigens, which is similar to the process of making CAR-T cells. Most studies have been successful in using lentiviral or retroviral-based transduction to make NK cells express stable and sustained CARs. Other methods of delivery, such as transposon systems and mRNA electroporation, have also been utilized [21,22,23,24]. The signaling domains of CAR-NK cells generally look very similar to those of CAR-T. The CAR structure consists of a fusion of CD3ζ with one or two TCR co-stimulatory molecules such as CD28, 4-1BB, 2B4, DNAM1, and NKG2D. Among these TCR-T co-stimulatory molecules, 4-1BB, DNAM-1, 2B4, and NKG2D were also expressed as intrinsic activation receptors on NK cells.

The specific antigens recognized by CAR-NKs vary widely. In the case of hematologic cancers, CD19 remains the primary target. In addition, a variety of other antigens, including FLT3, CS1, CD38, CD4, CD5, and CD7, have been also used as targets [25,26,27,28,29]. In STs, antigens such as EGFRvIII, Mesothelin, HER2, GD2, GPC3, EpCAM, PSCA, CEA, CD122, c- MET, and others have been targeted by CAR-NK cells [30,31,32,33,34,35,36,37,38,39,40,41,42,43,44,45,46]. In addition to directly killing tumor cells by targeting tumor antigens, some studies have shown that CAR-NK cells can be used to remove suppressive immune cells such as myeloid-derived suppressor cells (MDSCs) and M2 tumor-associated macrophages (TAMs) from the tumor microenvironment (TME) [15,47]. The role of CAR-NKs in TME could be used to enhance the therapeutic efficacy against STs.

There are also reports that CAR-NK cells secrete pro-inflammatory cytokines and chemokines which can improve the infiltration and function of CAR-T cells. Based on these studies, future CAR-NK and CAR-T combination therapies can be anticipated.

### 3.4. Clinical Trials

To date, a total of 76 CAR-NK therapies have been completed, including Umbilical and Cord Blood (CB)-Derived CAR-Engineered NK Cells for B Lymphoid Malignancies (NCT03056339), the first-in-human trial of CD19-CAR-NK, which has now completed phase 2. Currently, a large proportion of investigational CAR-NKs in clinical trials are targeting CD19. In addition, studies targeting hematologic cancers using various targets such as CD33, NKG2D ligands, and BCMA are underway. 

Although none of the CAR-NK studies targeting STs have been completed, studies are underway for various ST patients, including ovarian tumor (NCT05410717) and colon cancer (NCT05213195). In this case, tumor-specific antigens include NKG2D ligands, HERP-3, B7-H3, PDL-1, and 5T4. Other ongoing clinical trials include Autologous NK cell-based immunotherapy (NCT02185781) for ALL patients and Allogenic NK cell-based immunotherapy (NCT02845999) for Gastrointestinal Carcinoma patients.

### 3.5. Limitations

Treatment with NK cells has irreplaceable advantages as mentioned above. However, there are still issues that need to be addressed to utilize NK cells, such as difficulty in genetically manipulating them, lack of persistence to overcome functional exhaustion, and difficulty in finding cancer-specific antigens due to the heterogeneity of cancer, which also reduces the efficiency of CAR-NK. In order to utilize NK cells more effectively, research is underway to increase the functionality of NK cells and to overcome their shortcomings by increasing their stealth, specificity, resistance to TME, homing, and persistence [48]. 

## 4. Mφ-Based Immunotherapy

### 4.1. Characteristics of Mφ

As one of the essential innate immune cells, when activated, the macrophage (Mφ) mediates phagocytosis to capture and kill pathogens and apoptotic cells. The Mφ induces efficient tissue regeneration by supplying growth factors or anti-inflammatory molecules, etc. [49]. They play an important homeostatic role as phagocytes that maintain normal organ function by removing invading pathogens and large amounts of harmful endogenous substances such as apoptotic cells, dying red blood cells, amyloid beta, and surfactant [49,50]. They are also highly heterogeneous cells that can rapidly change their function in response to local microenvironmental cues [49,51]. Insights for the development of Mφ-based cell therapies have focused on their notable actions, such as promoting tissue regeneration and eliminating cancer cells or pathogens [49]. Due to the characteristics of the Mφ, Mφ-based cell therapy is being developed against cancer as well as skin wounds, neurodegenerative disease, liver cirrhosis, ischemic heart failure, inflammatory bowel disease (IBD) promoting intestinal regeneration, and pulmonary alveolar proteinosis.

### 4.2. CAR-M (CCL19)

Infiltration of the Mφ into tumors, unlike CAR-T or CAR-NK, is usually abundant, thus potentially overcoming the barriers to treating STs with cell-based therapies so far [4,52]. In contrast to lymphocyte-based therapies, the Mφ easily locates and sustains in the tumor microenvironment [52,53]. This is an attractive advantage for the use of the Mφ as an ST-specific therapeutic agent. CARs offer flexible platforms to direct immune cell effector function to antigen-expressing tumor cells and could facilitate the anti-tumor ability of the Mφ [52]. Recent efforts for designing CAR-Ms have shown that the fundamental principles of designing CAR from the T cell field could also be applied to CAR-M biology [4,52,54].

A study by Pierini S et al. on two immunocompromised NOD scid gamma (NSG) mouse xenograft models has demonstrated that a single dose anti-HER2 CAR-M resulted in decreased tumor burden and increased overall survival in HER2-positive SK-OV-3 tumors [52]. They also have shown an immuno-competent allogeneic CAR-M model and suggested that murine CAR-M increased intra-tumoral T cell infiltration, NK cell invasion, DCs infiltration/activation, and TILs activation [52,55]. Of particular note, this study also showed, for the first time, that CAR-M cells are synergistic with a PD-1 blockade in a PD-1 monotherapy-resistant ST model [52,55]. Niu et al. similarly achieved CAR-M-induced tumor killing using CCR7-targeted CAR-M in the RAW264.7 cell line [52,56]. In a 4T1 breast cancer model, CAR-M extended survival and prevented metastasis to distant tissues. CAR-M recruited CD3 T cells and reduced PD-L1+ cells at the tumor site—confirming that the engineered Mφ was not the only driver of the anti-tumor response—and increased serum levels of the inflammatory cytokines IL-1β, IL-6, and TNF-α, demonstrating a systemic immune response [52,56]. Another advantage of CAR-M is that the efficacy of CAR-M can be further enhanced by co-administering immunotherapy or chemotherapy. For example, antibody-based immunotherapy relies on Mφ phagocytosis to stimulate the immune response and can be evaluated to augment CAR-M efficacy [52,57,58].

### 4.3. Mφ as Efficient Transporters

Therapeutic agents (such as CpG-ASO and cisplatin) including nanoparticles are loaded on the Mφ and are delivered to the target site. This nanoparticle loading utilizes the Mφ to work more effectively as transporters [59].

Currently, the strategy of loading substances that act specifically on various cancers into nanoparticles and delivering them to cancer cells is being used [60]. Research is also underway to improve the types and functions of nanoparticles delivered by the Mφ. Xi Cao et al. found that paclitaxel-loaded RANPs exhibited significantly improved cytotoxicity and cell death rates compared to albumin nanoparticles without membrane coating, with a significantly enhanced antitumor efficacy [61]. In addition to loading nanoparticles, the Mφ can also deliver substances through surface-anchoring engineering. In this method, materials such as IFN-γ were delivered to the cell by anchoring to the cell’s surface [62]. 

### 4.4. Clinical Trials

Eleven clinical trials have been registered for Mφ-based cell therapy. Most focused on adoptive transfer and ex vivo polarization of the Mφ, and two of them on CAR-M. These studies about Mφ-based cell therapy target a variety of diseases, including cardiomyopathy, osteonecrosis, limb ischemia, stroke, arterial disease, and chronic anal fissures, but no cell therapy for tumors has been registered and completed as a clinical trial [49]. As the trend of Mφ clinical trials suggests, Mφ-based cell therapies often target diseases associated with regeneration. Therefore, the effectiveness of Mφ-based therapies against cancer is expected to be enhanced in the future.

### 4.5. Limitations

The Mφ must be produced at a scale that reduces the cost of treatment before it can be used for cell therapy [49,63]. Manufacturing facilities need to produce the Mφ stably and consistently. More importantly, with the rapidly increasing development of allogeneic therapies, the Mφ must be obtained from a variety of sources, including hematopoietic stem cells [64]. It is imperative that genetic modifications are performed using stable and integrated protocols and that the Mφ is obtained from different sources.

Another consideration in using the Mφ is plasticity. The Mφ has the ability to change phenotype in response to its environment [49,65]. However, this characteristic imposes a major disadvantage for the Mφ as cellular therapeutics. To ensure that Mφ-based cell therapies consistently maintain the desired phenotype, a fundamental understanding of Mφ polarization is mandatory. One way to address this is to create a genetically ‘fixed’ Mφ to overcome phenotype duration uncertainty [66,67]. Recently, Wei et al. reported on the development of tumor-associated Mφ (TAM) polarization therapy in combination with PLGA-DOX (PDOX)-induced immunogenic cell death (ICD) for the treatment of cancer [68]. This strategy shows that combining tumor-associated Mφ polarization therapy with ICD induced by low-dose chemotherapeutic drugs can significantly improve the efficacy of immunotherapy [68]. However, further research is still necessary to lock down the polarization. Furthermore, updates in nanoparticle loading methods or surface-anchoring techniques are needed to increase the role of Mφ. Finally, the development of more efficient CAR-Ms will require the establishment of optimized CAR constructs, similar to the case for CAR-Ts and CAR-NKs.

## 5. DC-Based Immunotherapy

### 5.1. Characteristics of DCs

Dendritic cells (DCs), first discovered by Ralph Steinman and Zanvil Cohn in 1973, are innate immune cells that play an important role in mediating the innate immune response and triggering the adaptive immune response. They are considered to be the most capable antigen presenting cell (APC), with the ability to activate both naive and memory immune responses.

DCs are cells that are specialized in priming antigen-specific T cells after taking up tumor antigens. Thus, DCs can initiate new antitumor responses and are an essential objective in ongoing efforts to improve antitumor immunity.

### 5.2. DC-Based Vaccination

DC vaccine immunotherapy is an approved treatment method that harnesses the patient’s own immune system to eliminate metastatic hormone-refractory cancer [69]. While the T cells described previously are powerful live drugs, the proportion of available tumor-specific T cells tends to be insufficient. To increase the number, or functionality, of the tumor-specific T cells, the patient’s own DCs can be activated to generate a therapeutic vaccine, ultimately priming the antigen-specific T cells to suppress the tumor. DC vaccination has been proven safe when used for a variety of tumor types [70]. Another advantage of DC vaccines is their availability for combination therapy. Currently, DC vaccines are used in league with a variety of therapies, including checkpoint blockade (CTLA-4, PDL-1, PD-1), cytokines (IL-2, IL-15, IL-17), TLR agonists (CpG, Poly-ICLC), oncolytic viruses, and STING agonists [70].

DC maturation is essential before DC vaccination because, unlike immature DCs, mature DCs can enhance the expression of costimulatory molecules, and produce cytokines or chemokines required for effective activation of the T cells to induce antitumor immunity [71] and to migrate into lymphoid tissues [72]. Therefore, in clinical trials, only mature, peptide-loaded DCs could induce an antigen-specific T cell response. Currently, two main methods are used for the maturation of DCs: ex vivo loading systems, in which immature DCs are isolated from the patient, matured, and reinjected into the patient, and in vivo targeting, in which inducers are introduced into the patient’s body to directly induce the priming of DCs already present.

#### 5.2.1. Ex Vivo Loading

Ex vivo loading is a method of isolating and maturing immature DCs such as monocyte precursors or CD34+ hematopoietic precursors from the patient. Isolated DCs are induced into mature DCs by adding adjuvants such as pro-inflammatory cytokines, CD40L, and TLR agonists. Matured DCs are loaded with antigens by adding peptides, proteins, or tumor lysates, delivering RNA through electroporation or transfection, transducing bacteria/viral vectors, or fusing with tumor cells. The antigen-loaded matured DCs are then reintroduced into the patient [69].

#### 5.2.2. In Vivo Targeting

Although ex vivo-prepared DCs have the advantage of being relatively close to the Mφ [73], the disadvantage is that their quality is highly dependent on the patient’s condition and the preparation process is cumbersome. An alternative approach is in vivo targeting, which directly targets DCs in vivo to induce their maturation.

In vivo targeting directly induces priming by adding combinations of antigens and adjuvants, antibody conjugate, polymeric particles, functionalized particles, etc., directly into the body. In this case, the tumor antigen must be delivered together with the tumor antigen adjuvant to induce the priming of DCs.

- Antigen and adjuvant combination

Recent advances in the field of nanotechnology have led to a proliferation of nanomaterials. For biomedical applications, liposomes, PLGA, nanoparticles, synthetic scaffolds, and carbon nanotubes could potentially be applied as delivery systems targeting DCs. Importantly, these approaches can deliver antigens and adjuvants together to target cells and promote the engraftment of robust DCs. The composition of the particles, their size and the charge of the delivery system are highly related to the efficiency of delivery and determine the uptake by specific types of cells. Therefore, exploiting the different physicochemical properties of different biocompatible materials could lead to selectively targeting specific subtypes of DCs. Recent studies have described some efficient liposomal vaccines [61,74]. Due to their charge and composition, these liposomes preferentially targeted the spleen and efficiently activated different types of DCs to induce a sustained anti-tumor T cell response.

- Antibody conjugate

However, co-administration of non-linked adjuvants cannot ensure that all cells targeted by the antibody conjugate are adequately activated. In addition, antigen-presenting cells (APCs) that do not present the desired antigen can be equally strongly activated, leading to unwanted responses to self-antigens [75]. To compensate for these drawbacks, antibody-antigen conjugates have been developed to induce more specific anti-tumor immunity. Antibody conjugates are a combination of an antigen and an adjuvant, with the advantage that the antigen and adjuvant can directly target the same target cell.

- Polymeric particle

The use of macroscopic three-dimensional scaffolds to recruit DCs into sites with high doses of antigen and adjuvants is another strategy to increase the efficacy of DC activation. The use of macroscopic three-dimensional scaffolds to recruit DCs into sites with high doses of antigen and adjuvants is another strategy to increase the efficacy of DC activation. Due to the rapid development of new materials engineering, there is an ongoing effort to use more advanced nanoparticles to enhance anticancer immune responses [76].

- Functionalized particle

Advances in nanotechnology could be applied to functionalize nanomaterials with DC-targeting antibodies for the delivery of cell-specific immunogenic cargo.

### 5.3. Clinical Trials

There have been more than 80 registered clinical trials of DC vaccines. Among these, more than 13 have completed phase 2 clinical trials, most of which have demonstrated safety. These trials have been conducted in the setting of hematological malignancies, prostate cancer, and other various cancers (NCT00345293, NCT02528682, NCT00970203). Combination therapies with various substances such as IL-2, Ipilimumab, autologous tumor lysate, yeast cell wall particles, etc., were also done (NCT00085436, NCT01302496, NCT02678741). Despite being at the forefront of immune cell therapies, DC vaccines have not shown high success rates in clinical trials [77]. Due to the low success rate, the number of clinical trials applying DC vaccines for anti-cancer therapy has decreased, and recent clinical trials are mainly focused on evaluating the safety and potential side effects of DC vaccine therapy [77]. While not yet showing significant efficacy, the high safety profile of DC vaccines makes them a promising cell therapy.

### 5.4. Limitations

#### 5.4.1. Lower Cost and Higher Quality for DC Vaccine Generation

There has been great promise and success in several preclinical studies targeting DCs in vivo for cancer vaccination. However, to date, large-scale GMP manufacturing of clinical nanomaterials for cancer vaccination has been well known to be difficult, time-consuming, expensive, challenging, and therefore requires a dedicated infrastructure. There are also challenges in ensuring the quality of patient-derived cell-based therapy products. Therefore, creating a DC vaccine with a guaranteed high quality while reducing costs is a major future goal.

#### 5.4.2. Demand for a Fundamental Understanding of DC-Related Immunology

Many patients who receive DC vaccines develop new T cell reactions; however, these often do not translate into eventual clinical responses. One possible explanation is that the immunosuppressive environment in vivo alters the viability and function of DCs and jeopardizes their ability to prime T cells. To improve the effectiveness of DC vaccines, it is essential to understand the organic relationship between injected DCs and the immunosuppressive environment.

Furthermore, while the mechanism of CD8+ T cell activation is relatively well understood, that of CD4+ T cells is not. Understanding the relationship between CD4+ T cells and DCs is fundamentally important to drive the activation of the immune system.

#### 5.4.3. Development of a Personalized DC Vaccine

The advantage of DC vaccines is that they could be used in a variety of combination therapies to personalize treatment for each patient. Patient-specific combinations could include combinations of tumor antigens and adjuvants delivered together, functionalized nanomaterials, and combinations of DC vaccines. While DCs are already one of the most widely used immunotherapies [77], further research will help to establish them as a safe therapeutic strategy while inducing robust and sustainable CD8+ and CD4+ T cell responses. Combinations with other therapies such as checkpoint blockade, cytokines, TLR agonists, oncolytic viruses, STING agonists, etc., could also be considered.

## 6. Conclusions

Cell-based immunotherapy has brought about a new phase of cell therapy thanks to various research and clinical trials. Unlike conventional chemotherapy, its high target recognition, efficiency, and low side effects have greatly contributed to improving the survival rate of cancer patients. However, it still requires much more effort for the application to actual patients. The advantages and disadvantages of using each cell type as an immunotherapeutic agent have been described above. There are common challenges that need to be addressed regardless of the type of immune cells.

First is the optimization and stabilization of the cell engineering process. Autologous cell (T, NK, Mφ, and DCs) transfer requires more research and development to obtain sufficient cells for treatment. The fact that the efficiency of the treatment depends on the patient’s condition also needs improvement. The use of cells directly from the patient also requires long manufacturing time and high cost. Although NK cells that could be transferred by allogeneic cell transfer can compensate for the shortcomings listed above, it is necessary to constantly study which NK cell from what source is most effective and to improve their durability and efficiency.

In addition, a breakthrough cell therapy for STs has not been established yet. While some cell therapies are going through clinical trials targeting STs, none have yet received FDA approval. There is a need for understanding the intrinsic connection between the TME and immune cells and also applying it to the development of cell therapies. While many challenges remain, the use of immune cells in anti-cancer therapy is clearly attractive and, with further research, should make a major contribution to the fight against tumors.
